# Feasibility of an Electronic Survey on iPads with In-Person Data Collectors for Data Collection with Health Care Professionals and Health Care Consumers in General Emergency Departments

**DOI:** 10.2196/resprot.5170

**Published:** 2016-06-29

**Authors:** Shannon D Scott, Lauren Albrecht, Lisa M Given, Danielle Arseneau, Terry P Klassen

**Affiliations:** ^1^ University of Alberta Faculty of Nursing Edmonton, AB Canada; ^2^ Charles Sturt University School of Information Studies New South Wales Australia; ^3^ University of British Columbia Faculty of Medicine Vancouver, BC Canada; ^4^ Children’s Hospital Research Institute of Manitoba Winnipeg, MB Canada

**Keywords:** survey development, electronic survey, survey implementation, needs assessment, pediatric emergency medicine

## Abstract

**Background:**

Translating Emergency Knowledge for Kids was established to bridge the research-practice gap in pediatric emergency care by bringing the best evidence to Canadian general emergency departments (EDs). The first step in this process was to conduct a national needs assessment to determine the information needs and preferences of health professionals and parents in this clinical setting.

**Objective:**

To describe the development and implementation of two electronic surveys, and determine the feasibility of collecting electronic survey data on iPads with in-person data collectors in a busy clinical environment.

**Methods:**

Two descriptive surveys were conducted in 32 general EDs. Specific factors were addressed in four survey development and implementation stages: survey design, survey delivery, survey completion, and survey return. Feasibility of the data collection approach was determined by evaluating participation rates, completion rates, average survey time to completion, and usability of the platform. Usability was assessed with the in-person data collectors on five key variables: interactivity, portability, innovativeness, security, and proficiency.

**Results:**

Health professional participation rates (1561/2575, 60.62%) and completion rates (1471/1561, 94.23%) were strong. Parental participation rates (974/1099, 88.63%) and completion rates (897/974, 92.09%) were excellent. Mean time to survey completion was 28.08 minutes for health professionals and 43.23 minutes for parents. Data collectors rated the platform “positively” to “very positively” on all five usability variables.

**Conclusions:**

A number of design and implementation considerations were explored and integrated into this mixed-mode survey data collection approach. Feasibility was demonstrated by the robust survey participation and completion rates, reasonable survey completion times, and very positive usability evaluation results.

## Introduction

The Translating Emergency Knowledge for Kids (TREKK) project aims to bridge the research-to-practice gap in pediatric emergency medicine, and reduce variable emergency care, by ensuring that practitioners in general emergency departments (EDs) have access to and apply the latest research evidence in their pediatric practice [1]. The first phase of the TREKK project (the *Needs Assessment*) surveyed health care professionals and parents seeking care for their children in general EDs, to determine information needs and preferences to guide the development of knowledge translation tools on key child health topics. Given the well-documented challenges of survey research [2], particularly in health research [3-9], specific factors were addressed in four survey development and implementation stages, including survey design, survey delivery, survey completion, and survey return. The aim of this research was to increase data quality (ie, increasing participation rates, reducing item nonresponse, and reducing dropouts) [10].

Despite a wealth of research, much debate remains regarding the superiority of electronic and/or mailed paper surveys [4,11]. Recent studies indicate that the future of survey research involves mixed-mode approaches (ie, two or more modes of administration including mail, web, telephone, and/or in-person) [12] and/or additional recruitment techniques to generate higher response rates [13-15]. However, further research is recommended to describe variations in survey content and administration, and effects on participation rates and data quality [12]. In this paper, we describe electronic survey development and implementation using iPads and in-person data collectors. We also detail the feasibility of this novel mixed-mode approach to survey research by providing survey response rate results, average length of time for survey completion, and the results of a usability evaluation with data collectors.

## Methods

### Survey Design

Given the complexity of the ED environment (ie, fast-paced, high volume, high acuity), traditional paper-based surveys were too cumbersome and resource-intensive for this study. An electronic survey was determined to be the most appropriate method to meet study timelines, due to ease of implementation across a large geographic area, and decreased administrative costs [5,12,14,16]. To be included in the survey, each participant was either a health care professional working in a participating general ED, or a parent seeking care for a child in the EDs. Participants were excluded if they were unable to read or write English or French.

Survey questions were developed using relevant research literature [3] and in consultation with content experts in pediatric emergency medicine, nursing, and information science. The surveys underwent several iterations, and face validity was determined through team meetings and pilot testing within the research team. Both surveys were developed in English and translated into French. The *Health Care Professional Needs Assessment* survey collected demographic information, current information-seeking practices, information needs related to caring for children in the ED, and preferences for receiving new information related to caring for children in the ED (Multimedia Appendix 1). The *Parent Needs Assessment* survey collected demographic information, information about the current visit to the ED, and health information needs and preferences (Multimedia Appendix 2).

In addition to survey content, six features affecting response rate of web-based surveys were considered: (1) general format, (2) length, (3) disclosure of survey progress, (4) visual presentation, (5) interactivity, and (6) question/response format [17]. A screen design format was selected to display one question per page, as this design has been shown to have lower item nonresponse than scrolling designs [17]. Much thought was given to survey length, and the surveys were constructed to achieve an average survey completion time of approximately 20 minutes. This target aimed to mitigate busyness as a barrier to health care professional participation [18], and to maximize the opportunity for parents to complete the survey in the waiting room before being brought into an examination room. Disclosure of survey progress has shown limited effect on response rates, so we did not incorporate this feature in our surveys [17]. In terms of visual presentation of the survey, a plain visual presentation approach with selective use of color was used, based on research indicating higher completion rates and later dropouts using this design [17]. *Sans serif* font was selected for ease of readability on a screen [19], questions and responses were numbered, and bolding, shading, italics, and color were used in a consistent fashion, with the aim to enhance understandability. Arrows were used to direct participants to subsequent screens. Interactivity was also incorporated, as it has been linked to lower item nonresponse [17]. This feature included automatic jumps to questions based on previous answers, and a *missing data* message was displayed when an item was left blank; however, responses were not forced due to the association of this option with higher dropout rates [17]. Four question/response formats were used throughout the surveys, and included single and multi-touch responses with radio buttons, sliding scales, and drag and drop boxes in which responses could be dragged to a new column and rank ordered (Multimedia Appendix 3).

### Survey Delivery

Consistent hardware was used at each site to streamline training and mitigate technological issues in survey delivery [5]. iPads were selected as the most effective survey delivery and data collection device because of their functionality and participant preference. iPads are lightweight devices that are easy to transport and hand to participants. The iPad interface is user-friendly and the touch screen technology, which has been shown to reduce mean time for patients completing questionnaires [20], enabled new and interesting approaches to survey question design [21]. In addition, previous research has demonstrated that both health care professionals and parental respondents preferred participation on a tablet compared to paper-based surveys [22-25], and found these devices easy to use [23,25]. Furthermore, electronic tablets have been shown to be a viable method of collecting patient self-report data in pediatric waiting rooms [26] and in rural settings [24].

### Survey Completion

External validity has been identified as a methodological issue of concern in survey research [3-5,27,28]; we attempted to address this issue by having in-person data collectors accompany the technology while conducting on-site recruitment. Census sampling aimed to recruit all health care professionals, and convenience sampling was used to recruit parents. The protocol for recruitment involved approaching all staff and parents to introduce TREKK and determine study eligibility. iPads were provided to interested parties to review the electronic consent form; once consent was indicated, participants automatically proceeded to the electronic survey. Data collectors were available throughout survey recruitment and completion to answer questions and assist with overcoming any technological barriers, including comfort with web browsers and touch screen technology. This consideration was based on research suggesting that the amount of contact and length of time in the field are important factors in health care professional response rates [4]. Surveys could be kept open for any length of time and were closed when the *Submit* button was indicated at the end of the survey, or the browser window was closed. The survey was designed with this flexibility, as data collection occurred in unpredictable and busy EDs.

### Survey Return & Technical Issues

The electronic survey platform incorporated synchronous and asynchronous data collection capability, meaning that data could be collected online and automatically uploaded to a secure server when a wireless connection was present, and data could also be collected offline, safely stored on the device, and later uploaded to a secure server once a wireless connection was available. This feature was particularly important in the ED setting, as many hospitals do not provide wireless internet and 3G/4G connectivity is limited or non-existent in rural and remote regions. This approach also addressed previously identified security concerns with cloud-based data storage [29,30]. Automatic data upload also eliminated the need for data entry, thus reducing the potential for error, and allowed the research team to monitor data quality via a secure, password-protected portal to provide feedback or additional training to data collectors. This functionality required enhanced device security; however, iPads are equipped with the appropriate security features to meet this need, including passcodes and restrictions to limit access and usage, encryption to protect information stored on the device, GPS technology to track the device, and remote and automatic data wiping capabilities (in the event that the device is lost or stolen) [31].

### Usability Evaluation of Data Collection Platform

A usability evaluation was conducted with data collectors using an anonymous 20-question electronic survey. The content of the survey was theoretically informed on the basis of a small-scale review of previous studies employing electronic platforms for data collection and the National Institutes of Health’s Usability Guidelines [32]. These guidelines outline key features of visual design and user experience, including interactivity, portability, innovativeness, security, and proficiency. The usability survey ranked features of the electronic survey and iPad on a five-point scale according to how positively or negatively these features affected their experience collecting data for the *TREKK Needs Assessment* (Multimedia Appendix 4). Face validity was determined via team meetings and pilot testing within the research team.

## Results

### Survey Participation & Completion Rates

**Figure 1:**
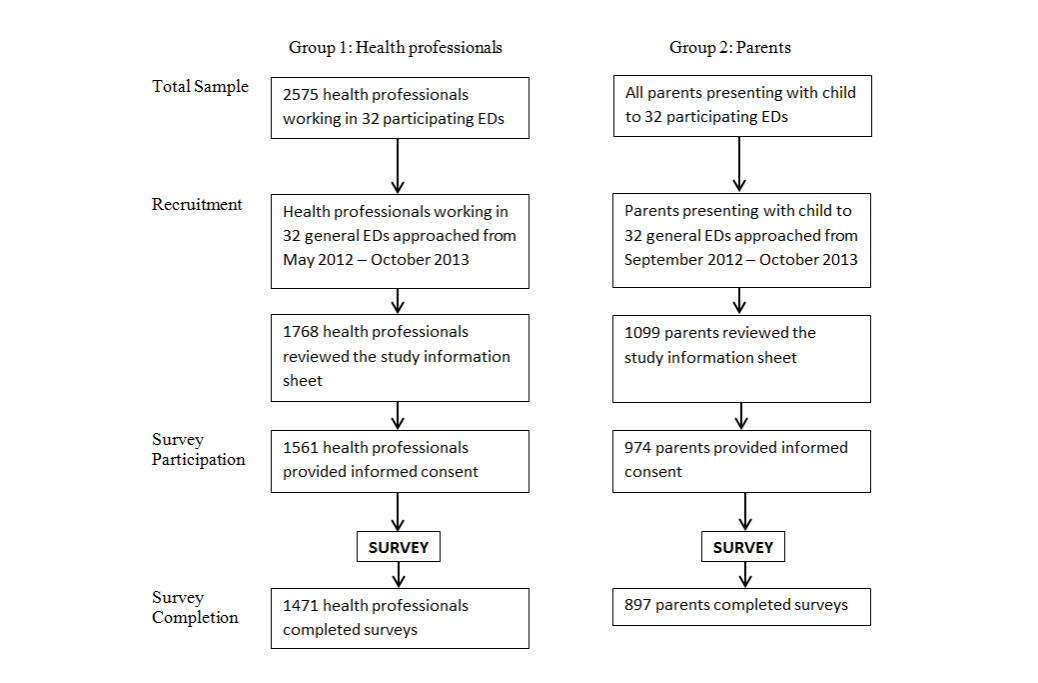
Survey recruitment, participation & completion.

The recruitment rate for health care professionals was 68.66% (1768/2575) and participation rate was 60.62% (1561/2575), and among participants the survey completion rate was 94.23% (1471/1561). Among parents the recruitment rate could not be determined because the eligible population was dependent on people coming into the general EDs; however, the participation rate was 88.63% (974/1099) and among participants the survey completion rate was 92.09% (897/974). See Figure 1 for recruitment and participation details for both populations.

### Length of Time for Survey Completion

Of the 1471 health care professional surveys included in the final analysis, 79 surveys did not have a time stamp (ie, participants did not press *Submit* at the end of the survey) and were not included in the calculation of average survey completion time. Of the 1391 surveys with a time stamp, the mean time to survey completion was 28.08 minutes (standard deviation = 118.54 minutes). This estimate includes participants that left the survey open and returned to complete it at a later time (eg, 9 surveys were open for more than 1000 minutes).

Of the 897 parental surveys included in the final analysis, 25 did not have a time stamp and were not included in the calculation for average survey completion time. Of the 872 surveys with a time stamp, the mean time to survey completion was 43.23 minutes (standard deviation = 691.25 minutes). This estimate includes participants that left the survey open and returned to complete it at a later time (eg, 4 surveys were open for more than 1000 minutes).

### Usability Evaluation of Data Collection Platform

**Figure 2:**
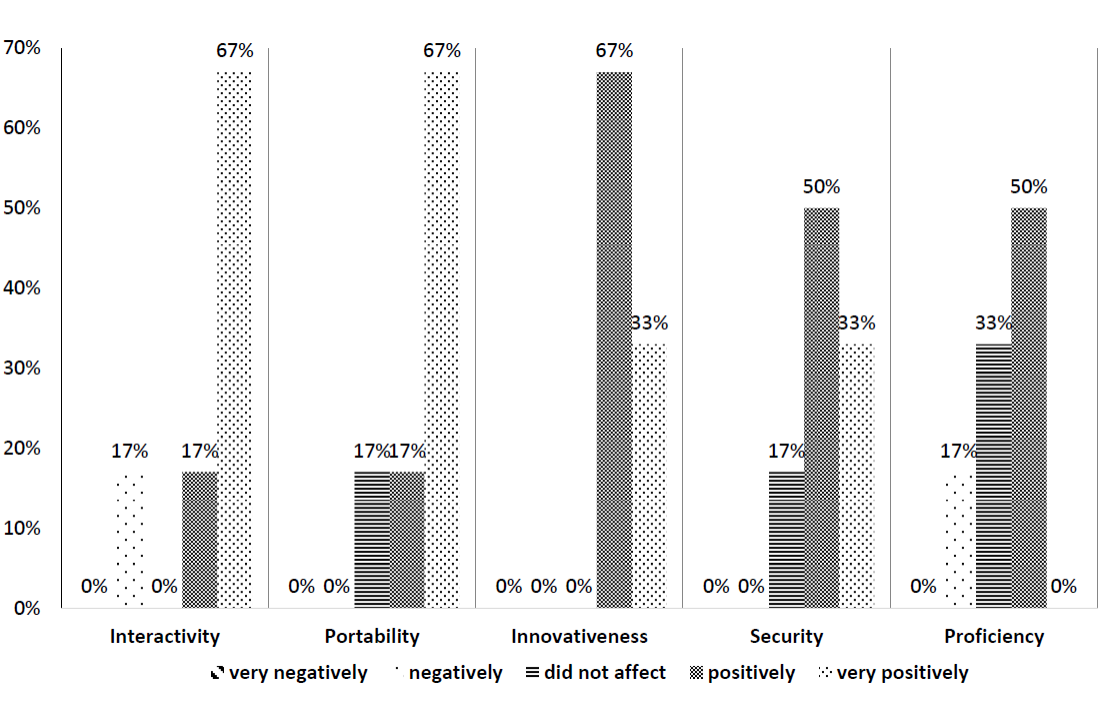
Data collectors' (n=6) ratings on 5 variables to evaluate the data collection platform.

Eight data collectors were approached to participate in the usability evaluation, six of whom agreed to participate and complete the survey (75% participation and completion rate). On a five-point scale, responses were largely *positive* (score=4) to *very positive* (score=5) on the five usability measures (see Figure 2). Additionally, respondents could enter free text information to describe, in detail, their perspectives on the strengths and weaknesses of iPads as a data collection tool. Respondents noted that the professional look and feel of the survey created a sense of trustworthiness and legitimacy of the research study, as demonstrated by the following excerpt, “With a unique online survey specific for TREKK, it appears more trustworthy as a legitimate research study, rather than having… paper surveys.” However, respondents illuminated some drawbacks to this approach, such as, “The only real negative of using the iPad relates to the survey participants [sic] level of comfort with technology, but not to such an extent that it affects participation - only initial comfort.”

## Discussion

Our findings make an important contribution to the web-based survey literature by addressing calls for research to examine web-based survey response and completion issues [10]. Generally, web-based surveys are correlated with low response rates [10], with estimates suggesting an average 10% decrease in response rates compared to traditional paper-based surveys. Given that 68.66% of health care professionals that were approached in our study reviewed the consent, and 94.23% of those who consented completed the survey, it is apparent that our mixed-mode approach mitigated these commonly accepted disadvantages of web-based surveys.

Utilizing data collectors to approach potential survey participants, and explain the study, eliminated the need for email or web-based invitations and completion reminders, and significantly enhanced survey participation and completion rates. We suggest that the addition of in-person data collectors offered the benefits of personal connection, and caused potential survey participants to make an active decision to participate in the study. In-person data collectors were also able to engage with potential participants and answer any questions the potential participants had about the study or the technology. Deploying a web-based survey without a mixed-mode approach allows potential participants to easily ignore electronic invitations to participate in survey research. With a mixed-mode approach, we were able to capitalize on the many benefits of web-based surveys, including improved data quality and the ability to immediately begin data analysis, while simultaneously mitigating previously reported downfalls of web-based surveys, including lower response rates and lower completion rates.

Vicente and Reis itemized six areas to consider when designing and implementing web-based surveys [17], and our usability findings support five of these recommendations (general structure, survey length, visual presentation, interactivity, and question/response format). Specifically, the usability findings collected by our in-person data collectors were largely *positive* to *very positive* for interactivity, portability, innovativeness, security, and proficiency. Free text responses further strengthened these findings by highlighting that the general structure, visual presentation, and question/response format of our survey helped to legitimize and enhance the credibility of our study. These findings highlight the importance of the investment of time and resources into survey design and implementation elements. Our participation and completion rates, and survey usability findings, are evidence that attention to survey design and implementation is strategic.

### Conclusions

This study provides strong evidence for the feasibility of a mixed-mode approach to survey data collection using iPads and in-person data collectors, based on strong response rates, reasonable survey completion times, and very positive usability evaluation results. This study also details survey development and implementation considerations that will be useful to survey researchers working with a variety of populations. Great potential exists for utilizing a mixed-mode approach for future survey research in clinical settings.

## Appendix

Multimedia Appendix 1 TREKK Needs Assessment Healthcare Professional Survey.

Multimedia Appendix 2 TREKK Needs Assessment Parent Survey.

Multimedia Appendix 3 Electronic survey question design.

Multimedia Appendix 4 TREKK Usability Survey with In-person Data Collectors.

## Abbreviations

ED: emergency department
TREKK: Translating Emergency Knowledge for Kids
