# Supplementary material for: Feasibility of an Electronic Survey on iPads with In-Person Data Collectors for Data Collection with Health Care Professionals and Health Care Consumers in General Emergency Departments
Source: JMIR Res Protoc. 2016 Jun 29;5(2):e139. doi: 10.2196/resprot.5170 (PMC4945822; doi:10.2196/resprot.5170)
Supplement: Multimedia Appendix 1 [file resprot_v5i2e139_app1.pdf]

Appendix A: TREKK Needs Assessment Healthcare Professional Survey

1. Please select your province by tapping the map below or choose from the list.
   - **a.** British Columbia
   - **b.** Alberta
   - **c.** Saskatchewan
   - **d.** Manitoba
   - **e.** Ontario
   - **f.** Québec
   - **g.** New Brunswick
   - **h.** Nova Scotia
   - **i.** Newfoundland and Labrador
   - **j.** Northwest Territories

2. Please select your location.

3. Please indicate your primary professional role.
   - **a.** Family physician (with an independent practice outside of the emergency department)
   - **b.** Emergency physician
   - **c.** Pediatrician
   - **d.** RN
   - **e.** LPN
   - **f.** Respiratory Therapist
   - **g.** Physiotherapist
   - **h.** Occupational Therapist
   - **i.** Registered Dietician
   - **j.** Social Worker
   - **k.** Pharmacist
   - **l.** Other: please specify (open text box)

4. How long have you worked in this profession?
   - **a.** __________years
   - **b.** Less than 1 year

5. Please indicate your gender.
   - **a.** Female
   - **b.** Male

6. Please indicate your age range.
   - **a.** <20 years of age
   - **b.** 20-24
   - **c.** 25-29
   - **d.** 30-34
   - **e.** 35-39
   - **f.** 40-44
   - **g.** 45-49

   - **h.** 50-54
   - **i.** 55-59
   - **j.** 60-64
   - **k.** 65-69
   - **l.** 70+ years of age

7. Please indicate the highest level of education you have achieved.
   - **a.** Diploma/Certificate
   - **b.** Bachelor’s Degree
   - **c.** Medical Degree
   - **d.** Master’s Degree
   - **e.** PhD

8. What type of unit do you work in most of the time?
   - **a.** Own practice
   - **b.** Emergency department
   - **c.** General medicine
   - **d.** Pediatric
   - **e.** General surgery
   - **f.** General medical surgical
   - **g.** Critical care
   - **h.** Medical specialty
   - **i.** Obstetrics
   - **j.** Operating room/recovery room
   - **k.** Surgical specialty
   - **l.** l. Other: please specify (open text box)

9. How long have you worked in this hospital?
   - **a.** __________years
   - **b.** Less than 1 year

10. What is your employment status in this hospital?
    - **a.** Full-time
    - **b.** Part-time
    - **c.** Casual
    - **d.** Not applicable (i.e., fee-for-service)

11. How frequently do you work in the emergency department?
    ___________ average shifts/month

12. How do you normally find information you need in order to work with children seeking care in the emergency department? *Drag the options from the “Do not use” column and place them in rank order in the “Use” column. Options can be re-ordered in the “Use” column.*
    - **a.** Academic or professional journals/articles
    - **b.** Internet search engine (e.g., Google)
    - **c.** Websites with medical/health focus (e.g. Up to Date)

   - **d.** Social media tools (e.g., Twitter, Facebook, etc.)
   - **e.** Printed resources (e.g., textbooks, brochures)
   - **f.** Talking with colleagues
   - **g.** Professional development opportunities (e.g., conferences, in-services, lunch & learns)
   - **h.** Other: please specify (open text box)

13. How would you assess your abilities to find, assess and use reliable clinical information/evidence to provide the best care to children in the emergency department? *Drag the pointer along the 10-point sliding scale from excellent ability to poor ability.*
   - **a.** Ability to locate information/evidence
   - **b.** Ability to assess information/evidence found
   - **c.** Ability to use information/evidence in practice

14. How effective are (response from Q. 11) that you typically use to find information you need in order to provide care to children in the emergency department? We have defined effectiveness as a combination of authority, accuracy, coverage, currency, and objectivity. *Drag the pointer along the 10-point sliding scale*
   - a. Authority: 1- Reputable and respected to 10- Unknown or unauthorized
   - b. Accurate: 1- Reliable and error free to 10- Not accurate
   - c. Coverage: 1- Thorough and detailed to 10- Incomplete or missing information
   - d. Current: 1- Recently updated to 10- Out of date
   - e. Objective: 1- Factual and unbiased to 10- Incorrect or biased

15. How many hours/week do you spend reading and/or finding information (e.g., in print, online, etc.) to help you provide care to children in the emergency department?
   __________average hours/week

16. How many hours/week do you spend reading and/or finding information (e.g., in print, online, etc.) to help you do your job? (not specific to children's health care)
   _____________ average hours/week

17. Which electronic devices do you use to look for new information **at work**? *Drag the options from the "Do not use" column and place them in rank order in the "Use" column. Options can be re-ordered in the "Use" column.*
   - **a.** Desktop computer
   - **b.** Laptop
   - **c.** Tablet (e.g., iPad, Blackberry PlayBook, Galaxy Tab, Kindle Fire, etc.)
   - **d.** Smartphone (e.g., iPhone, Blackberry, Android)
   - **e.** Other (please specify)_______ Use the least to look for new information

18. Which electronic devices do you use to look for new information **at home**? *Drag options from the "Do not use" column and place them in rank order in the "Use" column. Options can be re-ordered in the "Use" column.*
   - **a.** Desktop computer
   - **b.** Laptop

   c. Tablet (e.g., iPad, Blackberry PlayBook, Galaxy Tab, Kindle Fire, etc.)
   d. Smartphone (e.g., iPhone, Blackberry, Android)
   e. Other (please specify)__ Use the least to look for new information

19. Do you have Internet access at work?
   a. Yes
   b. No

20. Do you currently have the information you need to provide the best care to children in the emergency department? *Drag the pointer along the 10-point sliding scale from all information to no information*
   a. <u>Clinical Information</u>: Medical and/or health information needed to provide care to children
   b. <u>Patient & Family Support Information</u>: Information about how to interact & communicate with children receiving care and their families
   c. <u>Hospital Environment Information</u>: Information about the logistics of the care environment, such as policies and procedures, resources, physical space, etc.

21. What type of information do you need (that you currently do not have) in order to provide the best care to children in the emergency department? *Drag options from the "Do not need this information" column and place them in rank order in the "Need this information the most/Need this information the least" column. Options can be re-ordered in the "Need this information the most/Need this information the least" column.*
   a. Evidence-based clinical practice guidelines/pathways
   b. Evidence-based information about new diagnoses, treatments
   c. Protocols and currently accepted treatments for commonly seen conditions
   d. Summaries of new drugs (e.g., doses, frequency, etc.)
   e. Strategies for talking with parents about their child's illness/condition
   f. Strategies for talking with children about their illness/condition
   g. Child development information to guide patient interaction
   h. Strategies for dealing with children and families under stress/in crisis
   i. Strategies for explaining the hospital environment/logistics to children and families
   j. Explanation or documentation of emergency department specific procedures, policies and/or protocols
   k. Explanation or documentation of emergency department resources (e.g., computers, printed material, etc.)
   l. Other: please specify (open text box)

22. Do you require more clinical information about any of the following childhood conditions in order to provide the best care to children in the emergency department? *Drag options from the "Do not need more information" column and place them in rank order in the "Need more information the most/Need more information the least" column. Options can be re-ordered in the "Need more information the most/Need more information the least" column.*
   a. multi-system trauma
   b. severe head injury
   c. status epilepticus

d. meningitis
e. pneumonia
f. croup
g. bronchiolitis
h. asthma
i. fever
j. sepsis
k. urinary tract infection
l. septic joint
m. osteomyelitis
n. fractures (e.g., casting, reductions, procedural sedation)
o. laceration repair
p. reduction dislocated joint
q. nail repair
r. otitis media
s. cellulitis
t. gastroenteritis (dehydration)
u. strep pharyngitis
v. appendicitis
w. bowel obstruction
x. intussusception
y. SVT
z. first presentation of congenital heart defects
aa. diabetic ketoacidosis
bb. adrenal crisis
cc. congenital adrenal hyperplasia
dd. hematuria
ee. proteinuria
ff. hypertension
gg. jaundice
hh. neonatal hyperbilirubinemia
ii. hepatitis
jj. Other: please specify (open text box)

23. For what age range are you seeking additional information on (childhood condition from Q. 21) (check all that apply)?
    a. <1 year of age
    b. 1-2
    c. 3-5
    d. 6-8
    e. 9-11
    f. 12-15
    g. 16-18

24. Please indicate the areas where you require additional information (on childhood condition from Q. 21) (check all that apply).

a. Assessment of (childhood condition)
b. Diagnosis of (childhood condition)
c. Treatment of (childhood condition)

25. How would you like to receive new information about providing care to children in the emergency department? *Drag options from the "Would not like" column and place them in rank order in the "Would like most/Would like least" column. Options can be re-ordered in the "Would like most/Would like least" column.*
   a. Talking with colleagues (e.g., face to face meetings)
   b. Professional development opportunities (e.g., conferences, in-services, lunch & learns)
   c. Printed summaries (e.g., information sheets, brochures)
   d. Academic journals [q22_4_specify] Which one(s): ____________________
   e. SMS/Text message
   f. Email (e.g., electronic newsletter, listserv)
   g. App
   h. Facebook Group
   i. Twitter
   j. Website [q22_10_specify] Which one(s): (open text box)
   k. Other: please specify(open text box)
