# Supplementary material for: Feasibility of an Electronic Survey on iPads with In-Person Data Collectors for Data Collection with Health Care Professionals and Health Care Consumers in General Emergency Departments
Source: JMIR Res Protoc. 2016 Jun 29;5(2):e139. doi: 10.2196/resprot.5170 (PMC4945822; doi:10.2196/resprot.5170)
Supplement: Multimedia Appendix 2 [file resprot_v5i2e139_app2.pdf]

Appendix B: TREKK Needs Assessment Healthcare Consumer Survey

1. Please select your province by tapping the map below or choose from the list.
    a. British Columbia
    b. Alberta
    c. Saskatchewan
    d. Manitoba
    e. Ontario
    f. Québec
    g. New Brunswick
    h. Nova Scotia
    i. Newfoundland and Labrador
    j. Northwest Territories

2. Please select your location. *Touch region from a map to pull up a list of participating hospitals in that area.*
*Participants selected from a drop-down menu of hospitals

3. What is your gender?
    a. Female
    b. Male

4. What is your age range?
    a. <20 years of age
    b. 20-24
    c. 25-29
    d. 30-34
    e. 35-39
    f. 40-44
    g. 45-49
    h. 50-54
    i. 55-59
    j. 60-64
    k. 65-69
    l. 70+ years of age

5. What is the highest level of education you have completed?
    a. Elementary school completed, plus some high school credits (no diploma awarded)
    b. High school diploma or equivalent'
    c. Registered Apprenticeship or other trades certificate or diploma
    d. College, CEGEP, or other non-university certificate or diploma
    e. University certificate or diploma
    f. University undergraduate or professional degree
    g. University graduate degree (e.g., Master's, PhD)
    h. Other: Please specify (open text box)

6. How old is your child? _____ years_____ months

7. How many people usually live at the address where your child lives most of the time?

____ adults (18 years of age and older, including you)

____ children (under 18 years of age)

8. Are all adults living at this address related to your child?

a. yes

b. no

9. Are all children living at this address related to your child?

a. yes

b. no

10. How close is this emergency department to where your child lives most of the time?

a. in the same neighbourhood

b. in the same town/city

c. outside of the same town/city

11. What is the annual household income where your child lives most of the time?

a. under $25,000

b. $25,000- $49,000

c. $50,000-$74,000

d. $75,000 - $99,000

e. over $100,000

f. I prefer not to answer

12. Does your child have routine/regular health care check-ups (e.g., yearly) by a physician, nurse practitioner or a pediatrician?

a. Yes

b. No

c. Unsure

13. Why did you bring your child into the emergency department today? *Drag options to 1 (rank reasons) of 2 columns (leave items that do not apply)*

a. An ambulance was called because my child had a medical emergency

b. I thought my child required emergency medical attention

c. A healthcare professional (e.g., pediatrician, doctor, nurse) recommended that my child go to the emergency department

d. The after-hours message at the doctor’s office recommended that my child go to the emergency department

e. The telephone call-in service (i.e., HEALTHLINK) recommended that my child go to the emergency department

f. This is the closest place for medical attention

g. This is where I have come in the past

h. It takes too long to get an appointment with a family doctor

i. It takes too long to see a doctor at a walk-in clinic
j. My child does not have a family doctor
k. I do not go to walk-in clinics
l. I did not know where else to go

14. Did you bring your child to the emergency department today because of a known medical condition?
a. Yes
b. No

15. What is your child's known medical condition? (open text box)

16. Why did you bring your child to the emergency department today? *Drag options to 1 (rank reasons) of 2 columns (leave items that do not apply)*
a. Short of breath/ difficulty breathing
b. High temperature/fever
c. Throwing up
d. Diarrhea
e. Not eating/drinking
f. Fainting
g. Stomach pain
h. Sore ear/ear infection
i. Sore/swollen throat
j. Head injury
k.Broken/sprained bones
l. Cut/need stitches
m. Accident/injury
n. Other: please specify (open text box)

17. What's happening right now with your child's hospital visit?
a. Waiting to see a doctor
b. Met with a doctor
c. Left the emergency department without seeing a doctor
d. Left the emergency department after receiving care
e. Other: please specify (open text box)

18. What did the doctor do? *Check all that apply.*
a. Did more tests
b. Diagnosed your child
c. Gave your child treatment
d. Admitted your child to the hospital
e. Other: please specify (open text box)

19. How do you normally find health information that you need to care for your child? *Drag options to column 1 (rank items that they use) AND column 2 (place items that they do not use)*
a. Talking with friends, family members, etc.

b. Talking with trusted professionals (e.g., teacher, health professional, etc.)
c. At the library
d. Reading newspapers, magazines, etc.
e. Contacting specific agencies (e.g., local government, school board)
f. Internet search engine (e.g., Google)
g. Social media websites (e.g., Twitter, Facebook, etc.)
h. Smartphone Apps (e.g., WebMD mobile, Cures A-Z, iFirstAid, etc.)
i. Other: please specify (open text box)

20. Do you have all the information you need about bringing your child into the emergency department? *10-point sliding scale for each option (All information – No information)*
a. Medical and/or health information (e.g., symptoms, treatments, medicine, etc.)
b. When to come to the emergency department for medical attention
c. Strategies to comfort and/or distract your child in the emergency department
d. Strategies for dealing with your own feelings of stress, anxiety, concern, or uncertainty because your child is in the emergency department
e. Information about coming to the hospital (e.g., what to bring, how to get there, where to stay while the child is at the hospital, parking, how to access medical information, etc.)
f. Information about the emergency department (e.g., how to fill out forms, wait time, who will be caring for the child, etc.)

21. What type of information do you need (that you currently do not have) about being in the emergency department with this child? *Drag options to column 1 (rank items that they need) AND column 2 (place items that they do not need)*
a. Knowing when to bring your child to the emergency department
b. Reasons why your child is ill
c. Explanation about your child's illness (e.g., how long will it last, what will be the symptoms, how will I know if it is improving, etc.)
d.The treatment(s) that will be given to your child
e. How to care for your child after you leave the emergency department (e.g., how long should he/she stay home from school, when/where to get follow-up care, etc.)
f. How to comfort and/or distract your child(ren) while waiting to see a doctor
g. How to talk to your child about their illness/condition
h. How to talk to others (e.g., other family members, teachers, coaches, etc.) about your child's illness/condition
i. Information about the emergency department (e.g., how to fill out forms, wait time, who will be caring for your child, etc.)
j. Information about coming to the hospital (e.g., what to bring, how to get there, where to stay while your child is at the hospital, parking, how to access medical information, etc.)
k. How to deal with "life" (e.g., getting time off work, taking care of other children, contacting other family members, etc.)
l. Other: please specify (open text box)

22. How would you like to learn new information about your child's illness/condition? *Drag options to 1 (rank items that they like) of 2 columns (leave items that they do not like)*

a. In-person from healthcare professional(s) (e.g., doctor, nurse, etc.)
b. Over the telephone using a call-in service to speak with healthcare professionals (e.g., HealthLINK)
c. Verbally from family members, friends, etc.
d. Verbally from other parents of children with the same illness/condition
e. Reading storybooks about other families who have come to the emergency department for medical attention for the same illness/condition
f. Medical information sheets or brochures
g. Online through internet search engines (e.g., Google)
h. Online using specific medical/health websites
i. Online through social media websites (e.g., Facebook, Twitter)
j. Smartphone App
k. Email (e.g., electronic newsletter, listserv, etc.)
l. SMS/Text Message
m. At the library
n. Other: please specify (open text box)

23. What type of healthcare professional would you like to give you new information about the child's illness/condition? *Check all that apply.*
a. Pediatrician
b. Family doctor
c. Any doctor
d. Nurse practitioner
e. Nurse
f. Paramedic/EMT
g. Any healthcare professional
h. Other: please specify (open text box)

24. Did you look for information related to your child's health situation prior to coming to the emergency department today?
a. yes
b. no

<u>If the response to [q17] was YES, there were 3 follow up questions:</u>

25. Where did you look for this information? *Drag options to 1 (rank items that used) of 2 columns (leave items that they did not use)*
a. In-person from a healthcare professional (e.g., Family Doctor)
b. Over the telephone from a healthcare professional (e.g., Family Doctor, HealthLINK call-in service)
c. Verbally from family members, friends, etc.
d. Verbally from other parents of children with the same illness/condition
e. Internet search engine (e.g., Google)
f. Specific medical/health websites
g. Social media (e.g., Facebook, Twitter) websites
h. Smartphone App (e.g., WebMD mobile, Cures A-Z, iFirstAid, etc.)

i. At the library
j. Medical information sheet or brochure
k. Other: please specify (open text box)

26. What type of information did you look for? *Drag options to 1 (rank items that they looked for) of 2 columns (leave items that they did not look for)*

a. Information about specific symptoms shown by the child
b. General information about an illness/condition
c. Check to see if the issue is serious
d. Learn what to do next
e. Where to go for medical advice or attention (e.g., HEALTHLINK call-in service, walk-in clinics, hospitals, etc.)
f. Which hospital to go to
g. Location of the hospital and/or directions to get to the hospital
h. Where to park at the hospital
i. Nearby or all-night pharmacies
j. Child care options
k. Transportation options (e.g., bus schedule, phone number for taxi, etc.)
l. Other: please specify (open text box)

27. How helpful was the information you found about the child's health situation? *10-point sliding scale generated for each response given for #19 (very helpful – not at all helpful)*

a. Information about specific symptoms shown by the child
b. General information about an illness/condition
c. Check to see if the issue is serious
d. Learn what to do next
e. Where to go for medical advice or attention (e.g., HEALTHLink call-in service, walk-in clinics, hospitals, etc.)
f. Which hospital to go to
g. Location of the hospital and/or directions to get to the hospital
h. Where to park at the hospital
i. Nearby or all-night pharmacies
j. Child care options
k. Transportation options (e.g., bus schedule, phone number for taxi, etc.)
l. Other: please specify (open text box)

<u>If the answer to [q17] was NO, there was 1 follow-up question:</u>

28. Why not? *Drag options to 1 (rank reasons) of 2 columns (leave items that do not apply)*

a. Prefer to get information in the emergency department first
b. Did not have time because I was in a hurry to get to the emergency department
c. Did not have time because I had other "life" demands (e.g., work, taking care of additional children, etc.)
d. Did not know where to look for information
e. Did not know what questions to ask

f. Did not have access to any ways to look for information
g. Did not need any information
h. Other: please specify (open text box)
