# Supplementary material for: Feasibility of an Electronic Survey on iPads with In-Person Data Collectors for Data Collection with Health Care Professionals and Health Care Consumers in General Emergency Departments
Source: JMIR Res Protoc. 2016 Jun 29;5(2):e139. doi: 10.2196/resprot.5170 (PMC4945822; doi:10.2196/resprot.5170)
Supplement: Multimedia Appendix 3 [file resprot_v5i2e139_app3.pdf]

Appendix C: Electronic survey question design

Single-touch response

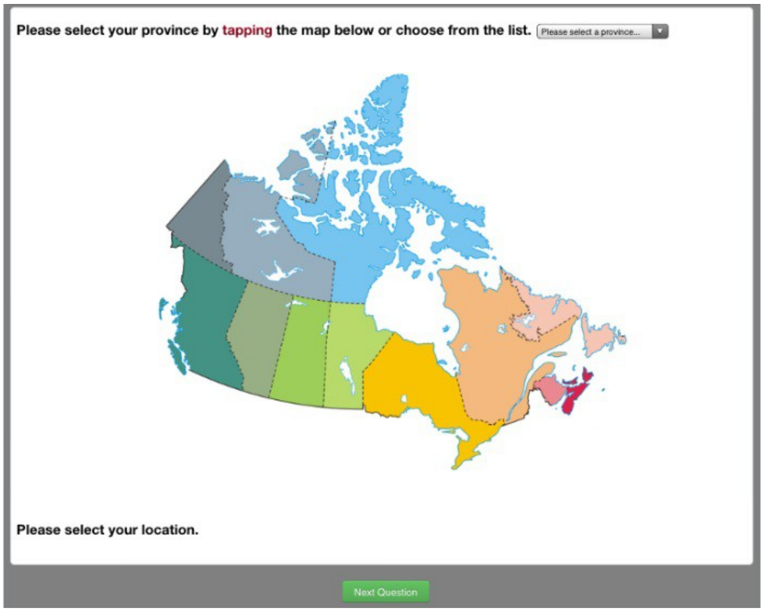

Sliding scales

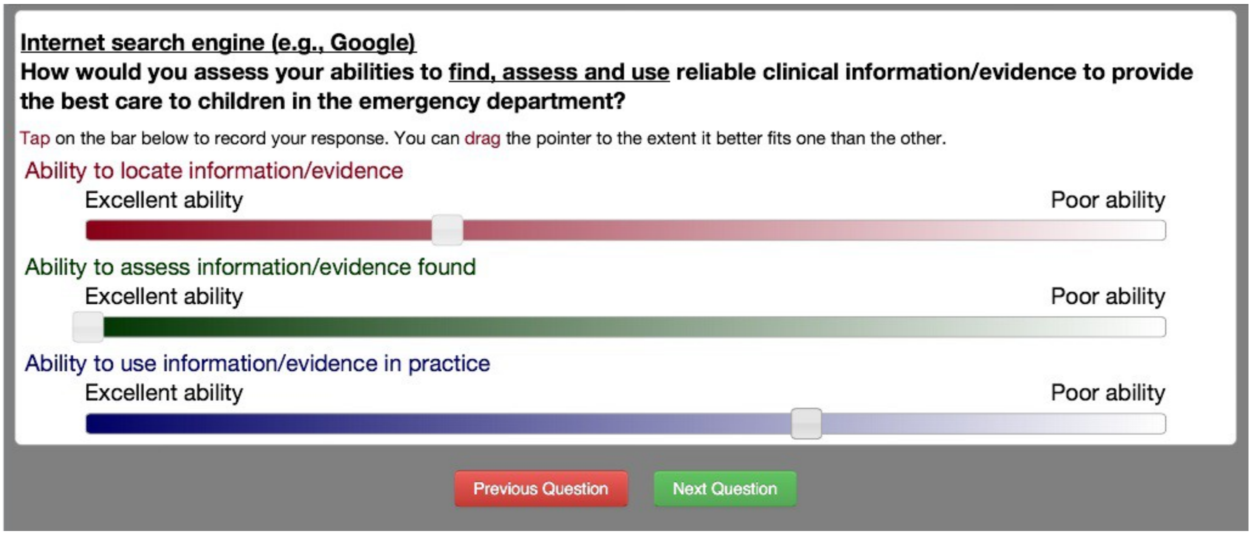

Electronic survey question design: Drag and drop boxes

How do you normally find information you need in order to work with children seeking care in the emergency department?

Drag the options from the 'Do not use' column and place them in rank order in the 'Use' column. Options can be re-ordered in the 'Use' column

Do not use to find information

- Academic or professional journals/articles
- Social media tools (e.g., Twitter, Facebook, etc.)
- Printed resources (e.g., textbooks, brochures)
- Talking with colleagues
- Other

Use the most to find information

1 Internet search engine (e.g., Google)

2 Websites with medical/health focus (e.g., Up to Date)

3 Professional development opportunities (e.g., conferences, in-services, lunch & learns)

Use the least to find information

Previous Question

Next Question
