# Supplementary material for: Feasibility of an Electronic Survey on iPads with In-Person Data Collectors for Data Collection with Health Care Professionals and Health Care Consumers in General Emergency Departments
Source: JMIR Res Protoc. 2016 Jun 29;5(2):e139. doi: 10.2196/resprot.5170 (PMC4945822; doi:10.2196/resprot.5170)
Supplement: Multimedia Appendix 4 [file resprot_v5i2e139_app4.pdf]

Appendix D: TREKK Usability Survey with In-person Data Collectors

1. How much previous research experience do you have, not including graduate student experience?
    a. Less than 1 year
    b. 1-5 years
    c. More than 5 years

2. What roles have you actively taken in your previous experience? *Check all that apply.*
    a. Project coordination
    b. Data collection
    c. Data analysis

3. What types of technology do you use in your daily life, personal or for work? *Check all that apply.*
    a. Cellphone
    b. Smartphone (ex: Blackberry, Android, etc.)
    c. iPhone
    d. iPod
    e. Desktop computer
    f. Laptop computer
    g. iPad (excluding the use in the TREKK project)
    h. Tablet (Samsung, HP, Sony, etc.)

4. How long have you been collecting data with an iPad in the TREKK project?
    a. Less than 1 month
    b. 1-3 months
    c. 3-5 months
    d. 6+months

5. The following list contains factors that may have positively or negatively affected your experience collecting data. *Please rate to what degree each factor positively or negatively affected your experience, using a 5 point Likert scale of very negatively to very positively:*
    a. Interactivity of iPad (touch screen, drag and drop, etc.)
    b. Portability of iPad
    c. Innovativeness or "novelty" of using iPad in research
    d. Security/ confidentiality of the iPad
    e. Level of proficiency of those completing the survey (parents or health care professionals)

6. Please list any other STRENGTHS or WEAKNESSES of using the iPads for data collection (open text box):

7. Would you recommend using iPads for data collection in future studies? Why or why not? (open text box)

8. Would you participate as a data collector in a future project using iPads? Why or why not? (open text box)
